# Viscoelastic Polyurethane Foam Biocomposites with Enhanced Flame Retardancy

**DOI:** 10.3390/polym16223189

**Published:** 2024-11-16

**Authors:** Grzegorz Węgrzyk, Dominik Grzęda, Milena Leszczyńska, Bartosz Nędza, Katarzyna Bulanda, Mariusz Oleksy, Joanna Ryszkowska, Ugis Cabulis

**Affiliations:** 1 Faculty of Materials Science and Engineering, Warsaw University of Technology, Wołoska 141, 02-507 Warsaw, Poland; grzegorz.wegrzyk.dokt@pw.edu.pl (G.W.); dominik.grzeda.dokt@pw.edu.pl (D.G.); bartosz.nedza.stud@pw.edu.pl (B.N.); joanna.ryszkowska@pw.edu.pl (J.R.); 2 Faculty of Chemistry, Rzeszów University of Technology, Powstańców Warszawy 6 Av., 35-959 Rzeszow, Poland; k.bulanda@prz.edu.pl (K.B.); molek@prz.edu.pl (M.O.); 3 Latvian State Institute of Wood Chemistry, 27 Dzerbenes Str., LV-1006 Riga, Latvia; ugis.cabulis@kki.lv

**Keywords:** viscoelastic polyurethane foams, renewable sources, natural filler, flame retardants, third-generation blowing agent, blackcurrant pomace

## Abstract

The growing demand for viscoelastic polyurethane foams creates a need for new sustainable raw materials that support cost-effective production while maintaining the desired material performance and fire safety standards. In this regard, our study aimed to develop viscoelastic polyurethane foam composites with reduced flammability and a high proportion of renewable raw materials. To achieve this, blackcurrant pomace, expandable graphite and a third-generation blowing agent were introduced to a viscoelastic polyurethane foam composition containing a reactive flame retardant in the formulation. The effects of the incorporated additives on the foaming process, flammability, chemical structure, cellular structure, thermal properties and physico-mechanical properties of the composites were determined. The results showed that the viscoelastic foam composite containing 30 php of blackcurrant pomace and 15 php of expandable graphite had a pHRRmax 52% lower than that of the reference material. The additional use of a blowing agent enhanced the flame-retardant effect of the materials, resulting in a 67% reduction in pHRRmax of the composite compared to the reference material. Moreover, the developed biocomposites exhibited promising limiting oxygen index values of 26–28%, compared to the 21% shown for the reference sample. Consequently, the best-performing biocomposites achieved the V-0 flammability rating according to the UL-94 standard. This study’s results indicate the composites’ high application potential due to their reduced flammability and the materials’ desirable physical and mechanical properties.

## 1. Introduction

The dynamic development of polyurethanes (PUs) is underway due to their versatile properties, resulting from a wide range of possible raw materials and compositions as well as manufacturing methods and conditions, which enable the formation of PU materials with a wide range of mechanical, chemical, physical and biological properties [1,2,3,4]. The global polyurethane material market was valued at USD 78.07 billion in 2023 and is projected to have an average annual growth rate (CAGR—Compound Annual Growth Rate) of 4.5% during 2024–2030. Foams account for the largest share of the polyurethane material market, which can be classified into flexible, rigid and semi-rigid foams [5]. The specific properties of flexible foams have led to their usage in industrial areas such as furniture, automotive and medical industries, where they are used to manufacture mattresses, car seats, cushions and orthopaedic insoles [6,7,8].

Due to the polymer matrix’s highly developed surface and chemical composition, foams are flammable materials [9,10]. The limiting oxygen index (LOI) of unmodified PUR foams is in the range of 16–18 (ISO 4589) [11]. Polyurethane foams decompose at temperatures above 200 °C, and the combustion results in the emission of toxic fumes and gases, including various chemicals, for example, nitriles, nitrogen oxides, phenylisocyanate, p-tolueneisocyanate, isoquinoline, o-benzodinitrile, xylene, benzene, biphenyl, naphthalene, carbazole and dimethylbiphenyl. Moreover, the concentrations of hydrogen cyanide and carbon oxides in the thermal degradation products of PU materials increase with temperature. Benzonitrile, which is the dominant non-volatile decomposition product, undergoes further decomposition, leading to the formation of HCN. The type and content of combustion products depend on the chemical structure of the polyurethane [12,13,14]. Limiting the flammability of foams requires introducing multidirectional solutions, both at the matrix material design level and using flame retardants [15]. Inexpensive and effective chlorine and bromine flame retardants have been widely used in reducing the flammability of plastics, but their use is now restricted in the US and EU member states due to the toxicity of the products formed during combustion, as well as the corrosive properties of the gases released. As a result, there is a growing need for alternative flame-retarding additives for PU, such as expandable graphite, phosphoric and nitrogenous compounds, nanofillers or aluminium and magnesium hydroxides, which are not toxic and are more environmentally friendly. However, their lower efficiency compared to chlorine and bromine flame retardants generates the need to use two to three times the amount of such fillers, which negatively affects the final parameters of the product [12,16,17]. The issue of reducing the flammability of polyurethane materials of their flammability is a major problem, and its effective solutions can be a decisive factor in the further development of this group of polymers.

Another important problem in the polyurethane foam market is the need to look for new, environmentally friendly raw material solutions [6,18,19,20]. One of the interesting and promising directions in line with the idea of a circular economy is the use of waste materials from the agro-food industry to produce polymeric materials [21]. The huge amount of waste generated during food production is an increasing challenge for manufacturers not only because of the very high disposal cost but also because of increasing environmental requirements [22,23]. According to the literature, the share of unused raw material in the production of fruit and vegetable juices is 30÷50%; in the production of vegetable oils, it is 40÷70%, and in the production of sugar from sugar beets reaches 86% [24]. Such waste is repurposed into animal feed and fertilisers, as well as utilised in the manufacture of distillates, flavours, dyes and pectic substances, and it is also subject to incineration with energy recovery [21]. A significant share of production waste is also subjected to composting and landfilling, which produce harmful agents, mainly gases such as carbon dioxide, methane, ammonia and hydrogen sulphide, affecting global warming [25]. The literature suggests that introducing natural fillers into PU compositions may disrupt the foaming process due to the effect of solid particles on the nucleation process and microbubble formation. In some cases, the increase in apparent density is disproportionate to its amount [26]. Changes in the foaming process can also result from the release of easily volatile substances from the filler, including water, which is a blowing agent in polyurethane foam synthesis [25,27]. Further limiting factors in the use of natural fillers as additives for PU foams include limited adhesion between the filler and the polymer matrix, disruption of the cell structure and deterioration of mechanical properties as well as increased flammability, which might result not only from the incineration of the filler particles but also from the changed pore structure of the polymer, facilitating the flow of air into the material [28]. However, waste from the agro-food industry, after proper preparation and selection of PU formulations, can be a valuable raw material for the manufacture of polyurethane foams due to the possibility of reducing the price of the material by using a low-cost waste filler, as well as obtaining the desired functional properties [29]. Fillers of plant origin can be used in the form of fibres or particles and obtained from seeds, hulls, shells and also fruits, leaves and stems of plants. As fillers of animal origin, feathers, hair or shells can be used [29,30]. A key factor in the profitability of producing composites with natural fillers is ensuring a reliable supply of consistently high-quality fillers, sourced from locations near the polymer processing plants [31].

The selection of foaming agents is also important in developing the polyurethane material market. Due to tightening environmental regulations, successive generations of porophores must follow strict standards. Regulations such as the Montreal Protocol and the REACH directive restrict the use of ozone-depleting substances and those with high global warming potential. New porophores are being designed to have minimal environmental impact and allow companies to comply with global standards and requirements, but research is needed to determine the impact of these additives on the structure and properties of foams.

The growing number of research studies addressing the impact of natural raw materials on flammability, as well as strategies for reducing the flammability of foams produced from these components, underscores the importance and relevance of this subject for the advancement of this material category. In the study conducted by Ahir et al. [32], the flammability of rigid foams formulated with polyol derived from hemp seed oil and microcrystalline cellulose was significantly mitigated by incorporating melamine at a high concentration of 75 parts per hundred of polyol (php). The investigation by Członka et al. [33] revealed that the utilisation of hemp shives impregnated with sunflower oil and tung oil in the synthesis of rigid polyurethane foams resulted in a reduction in the peak heat release rate, total smoke release and limiting oxygen index. However, it is noteworthy that these materials displayed a low renewable content, amounting to only 2 php, potentially constraining their sustainability profile. A research paper by Oliwa et al. [28] achieved a reduction in the flammability of viscoelastic foams through the application of expandable graphite but achieved an unfavourable increase of more than 40% in the apparent density of the composite.

Despite these significant contributions to the field, a research gap persists concerning the effective integration of natural raw materials, including organic fillers, with flame retardant additives to develop polyurethane foams that exhibit both high functionality and sustainable characteristics. To enhance the application potential of polyurethane materials derived from renewable resources, it is imperative to formulate composites that combine renewable fillers, such as agro-food waste, with flame-retardant additives while maintaining advantageous physical and mechanical properties.

To the best of our knowledge, this study is the first to explore the effect of using blackcurrant pomace, expandable graphite and a third-generation blowing agent with the trade name SOLKANE^®^ 365/227 (Solway, Neder-Over-Heembeek, Brussels, Belgium) on the properties of viscoelastic foams based on formulations containing the reactive flame retardant Exolit^®^ OP550 (Clariant AG, Muttenz (Basel-Landschaft), Switzerland). Incorporating a low-cost waste filler, such as blackcurrant pomace, aligns with the principles of a circular economy, improving both the environmental and economic value of the produced composites. To increase the safety of the use of the polyurethane composite with natural filler, the reactive flame retardant Exolit^®^ OP550 and expandable graphite were also used to produce the materials. SOLKANE^®^ 365/227 was used as an additional blowing agent. The research included an analysis of the foaming process, flammability, chemical structure, cellular structure, thermal properties and physical–mechanical properties of the materials produced.

## 2. Materials and Methods

The selection of formulations, additives and specific processing parameters and test conditions was based on consultations with industry partner Fampur Adam Przekurat, preliminary studies’ results and research results obtained in the work of Oliwa et al. [28].

### 2.1. Raw Materials for the Manufacture of Polyurethane Foams and Their Composites

The reference material (VEF) was produced using components A and B based on a commercially available system for producing viscoelastic polyurethane foams with the trade name Classic by Fampur Adam Przekurat. Component A consisted of the following substrates: Daltocel^®^ F442 (Huntsman, The Woodlands, TX, USA)—polyetherol with functionality 3, hydroxyl value 42 mg KOH/g, ethoxy (EO) group content approximately 76%; Daltocel^®^ F526 (Huntsman, The Woodlands, TX, USA)—polyetherol with functionality 3, hydroxyl value 126 mg KOH/g, EO group content over 70%; Rokopol^®^ F3600 (PCC Rokita SA, Brzeg Dolny, Poland)—polyetherol with functionality 3, hydroxyl value 48 mg KOH/g; Rokopol^®^ M1170 (Brzeg Dolny, Poland)—polyetherol with functionality 3, hydroxyl value 35 mg KOH/g, EO group content over 50%; Jeffcat^®^ DPA (Huntsman, The Woodlands, TX, USA), gelation catalyst—*N*-(3-dimethylaminopropyl)-*N*,*N*-diisopropanolamine; Jeffcat^®^ ZF10 (Huntsman, The Woodlands, TX, USA), blowing catalyst—*N*,*N*,*N*’-trimethyl-*N*’-hydroxyethyl-bisamino ethylether; Tegostab^®^ B4900 (Evonik Industries, Herne, Germany), surfactant—polyether modified polysiloxane; distilled water-blowing agent. In addition, Exolit^®^ OP 550 (Clariant International Ltd., Muttenz, Switzerland) was introduced into the system as a reactive flame retardant—non-halogenated phosphorus polyol with functionality 2, hydroxyl value 170 mg KOH/g, phosphorous content 16–18% at share 10 php. Ongronat 4040 (BorsodChem, Kazincbarcika, Hungary) was used as component B. Ongronat 4040 is mixture of 4,40-diphenylmethane diisocyanate, o-(p-isocyanarobenzyl) phenylisocyanate, polyisocyanate and polyphenylmethane; its functionality is 2, and it contains 32.4% NCO groups. The foams were produced with an isocyanate index of 90. The shares of the listed raw materials were set based on the recommendation of Fampur Adam Przekurat Company (Bydgoszcz, Poland).

The following additives were introduced to the foam:

Blackcurrant pomace (BC)—by-product of the agro-food industry supplied by AGROPOL Fruit and Vegetable Processing Plant, Potycz, Góra Kalwaria, Poland. The delivered pomace was ground in an impact mill and then dried at 70 °C for 18 h.

Expandable graphite EG399 (G)—flake graphite with a carbon content of min. 99%, expansion degree 250–400 mL/g, bulk density of 0.7 g/cm^3^ and grain size in the 400–1000 µm range, supplied by Sinograf, Toruń, Poland.

SOLKANE^®^ 365/227 (S)—a third-generation blowing agent supplied by Solvay (Brussels, Belgium). It is a non-flammable mixture of hydrofluorocarbons (1,1,1,3,3-pentafluorobutane and 1,1,1,2,3,3,3,3-heptafluoropropane in a weight ratio of 93:7) with no ozone-depleting potential.

### 2.2. Synthesis of the Polyurethane Foams and Their Composites

Considering the efficiency and cost of the manufacturing process, polyurethane foams were synthesised using a one-step method. The weighed components of component A, which consisted of polyols, catalyst, surfactants and blowing agents, were mixed using a mechanical stirrer until a homogeneous mixture was obtained (10 s, 3000 rmp). Next, powder additives G and BC were introduced into component A in the amounts listed in Table 1, and the system was again mixed using a mechanical stirrer (20 s, 3000 rmp). In the next step, component B was introduced and the system was stirred for 10 s at 3000 rmp, after which the mixture was immediately poured into an open rectangular polypropylene mould of 200 × 200 × 200 mm. After the synthesis process, the composites were annealed in a temperature chamber for 60 min at 70 °C, then seasoned for 3 days at room temperature. After seasoning, the materials were cut into samples required for the tests.

### 2.3. Methods

The characteristic times of the foaming process (start and growth time) were determined using an electronic stopwatch. They were measured from the moment the components were mixed until the beginning of the visible volumetric expansion of the reaction mixture (start time), and upon reaching the maximum foam height (growth time).

The shape and size of filler particles and foam cells were analysed using a Hitachi TM3000 SEM (Hitachi Group, Tokyo, Japan) with an accelerating voltage of 15 keV. All samples were coated with a layer of palladium and gold using a Polaron SC7640 sputter coater (Quorum Technologies Ltd., Laughton, UK) for 100 s at 10 mA before SEM imaging. Based on the obtained SEM images of the foams, the equivalent pore diameters were determined, with the analysis performed using ImageJ software v1.54k. Calculations included at least 50 pores for each foam variant.

Thermogravimetric analysis (TGA) was carried out using a TGA Q500 (TA Instruments, New Castle, DE, USA). Samples weighing 10 ± 0.5 mg were heated in a nitrogen atmosphere from room temperature to 600 °C at the rate of 10 °C/min. Data analysis was conducted with Universal Analysis 2000 software, version 4.7 A, by TA Instruments. Samples of 10 ± 0.5 mg were also measured in an air atmosphere from room temperature to 1000 °C at the rate of 10 °C/min to determine the appearance of char after burning. A KEYENCE VHX-970F digital microscope and a KEYENCE VHF-7020 camera (Keyence Corporation, Osaka, Japan) were used to analyse the surface microstructure of PUR foams after combustion.

The flammability tests were carried out using the cone microcalorimeter MLC from Fire Testing Technology Ltd. (East Grinstead, UK) equipped with a thermocouple. The samples, measuring 100 × 100 × 10 mm, were subjected to a heat flux of 25 kW/m^2^, and the distance from the ignition source was 25 mm (ISO 13927). On the basis of this study, the basic parameters determining the behaviour of the foams during burning were directly determined, such as heat release rate (HRR); peak heat release rate (pHRR); maximum peak heat release rate (pHRRmax); total heat released (THR); time to ignition (Ti); and residue after burning (Rc).

The oxygen index (LOI) was determined according to ISO 4589 using equipment from FTT Ltd. (East Grinstead, UK). Ten samples of 100 ± 0.5 mm × 10 ± 0.5 mm × 10 ± 0.5 mm were tested for each type of foam.

The UL-94 flammability class of the materials was determined using a chamber from FTT Ltd. (East Grinstead, UK). The measurements were performed in accordance with EN 60695-11-10, with horizontal and vertical sample beam positions and a methane-fed burner of 25 mm in height. The samples of 125 ± 5 mm × 13 ± 0.5 mm × 10 ± 5 mm were tested in the UL-94 test.

The differential scanning calorimetry (DSC) was conducted with a DSC Q1000 (TA Instruments, New Castle, DE, USA) using 5 ± 0.2 mg samples placed in hermetic aluminium pans. The procedure involved heating the samples at 10 °C/min, cooling at 5 °C/min and then reheating at 10 °C/min. The temperature and thermal effects of phase transitions were analysed using Universal Analysis 2000 TA software, version 4.7 A.

The Fourier transform infrared spectroscopy (FTIR) was carried out with a Nicolet 6700 spectrometer with an attenuated total reflection (ATR) accessory (Thermo Electrone Corporation, Waltham, MA, USA). Spectral data were collected over 64 scans in the 4000–400 cm^−1^ range. The results were analysed using OMNIC 8.2.0 software by ThermoFisher Scientific Inc. (Waltham, MA, USA).

The apparent density, compression set (50%, 22h at 70 °C) and rebound resilience of the foams were determined according to standards ISO 845, ISO 1856 and ISO 8307, respectively.

Compression tests were performed according to EN ISO 3386 on an Instron 5565 (Instron, Norwood, MA, USA) double-column test machine.

## 3. Results and Discussion

### 3.1. Analysis of the Foaming Process

The start and growth time analysis results indicate that modifying the systems with additives such as expandable graphite, blackcurrant pomace, and SOLKANE^®^ 365/227 affected the characteristic times of the foaming process (Table 2).

Start times increased after the introduction of solid additives, which may be due to increased viscosity of the systems. The higher viscosity of the containing blends reduces the mobility of the particles, which disrupts the foaming kinetics and slows the PU polymerisation rate. Adding organic/inorganic fillers may affect the proper stoichiometry of the reaction between isocyanate and hydroxyl groups of polyurethane systems. This effect was also observed in the work of other researchers after the introduction of such fillers as nutmeg [34], oak bark [35], wood fibres [36] and expandable graphite [28] into the PU system. Blackcurrant pomace contains organic acids, which may influence the synthesis process of the foam. As presented in the work of Ryszkowska et al. [25], with increasing acidity, the reaction rate of hydroxyl and urea groups and isocyanate groups decreases. Expandable graphite is obtained in a process where strong acids are used, the residues of which can also slow down the synthesis process. The introduction of an additional blowing agent into the VEF_G system in the form of SOLKANE^®^ 365/227 (VEF_G_S) resulted in a reduction in the start time of the foaming process, which was also observed in other publications [37]. The growth stage is associated with a change in the volume of the reaction mixture. New bubbles are no longer formed at this stage, but there is an intensive increase in the volume of bubbles formed at the creaming stage. The introduction of graphite into the VEF starting system and SOLKANE^®^ 365/227 resulted in a shorter growth time. The increase in viscosity of the system caused by the use of blackcurrant pomace led to an increase in the volume expansion time of the foams.

### 3.2. Microstructural Analysis

**Figure 1:**
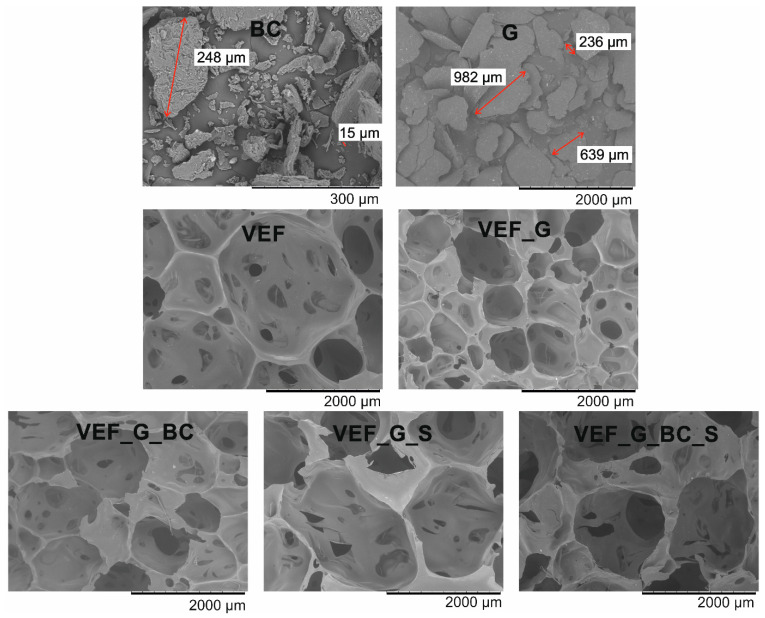
SEM images of the fillers and foams.

**Figure 2:**
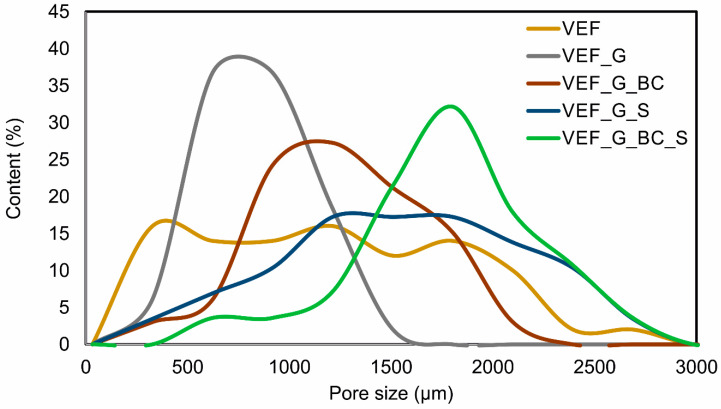
Pore size distribution in foams.

The morphology of the plant filler, expandable graphite as well as the changes in the cell structure of the foams caused by the introduction of the solid additives and additional blowing agents were visualised in images captured using a scanning electron microscope (Figure 1). The results of the cell size analysis are summarised in Figure 2 and Table 3.

Observations of the fillers performed using a scanning electron microscope indicate that the blackcurrant pomace particles obtained after grinding are characterised by a variety of shapes and sizes contained in the range of 15–600 µm (Figure 1). This is due to the processing of various parts of the plant: fruit peels, seeds, knobs, remains of inflorescences. Small particles with irregular shapes and spiral-twisted cellulose microfibrils were observed. Observations of expandable graphite performed using a scanning electron microscope confirm the information provided by the manufacturer, as irregularly shaped flakes with sizes in the 200–1000 µm range were observed.

The results of the pore size and shape analysis indicate that the VEF starting material was characterised by a high proportion of oval-shaped pores, where the average equivalent diameters were contained in the range of 327–2909 µm, and the average diameter value was 1354 µm. The introduction of expandable graphite into the system resulted in a reduction in the cell size range to the range of 411–1562 µm, with a predominance of cell sizes around 700–1000 µm, indicating that the expandable graphite particles acted as nuclei for new cells, resulting in the formation of a structure with a higher proportion of smaller pores. The addition of blackcurrant pomace to the VEF_G system (VEF_G_BC) resulted in an increase in cell size, which may be due to an increase in the viscosity of the system after the introduction of additional filler, as also indicated in the publications of other authors [38]. For the VEF_G_BC material, a predominant proportion of cells with a size of 800–1500 µm was observed. The introduction of an additional blowing agent, both to the VEF_G system (VEF_G_S) and the VEF_G_BC system (VEF_G_BC_S), resulted in a wider range of cell sizes and pores with greater shape irregularity.

### 3.3. Thermogravimetric Analysis

**Figure 3:**
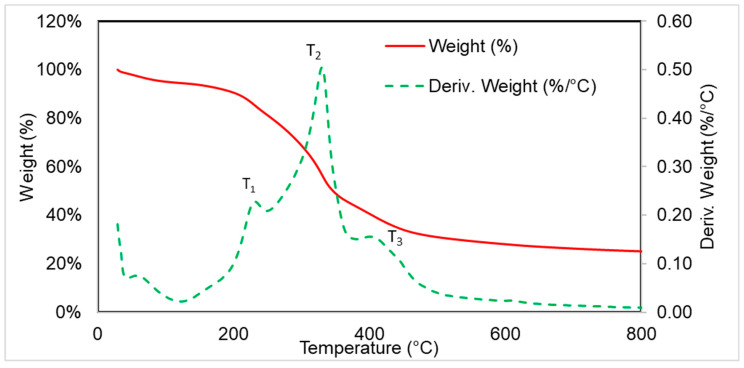
TG and DTG thermograms of blackcurrant pomace.

Figure 3 shows a graph obtained by thermogravimetric analysis of blackcurrant pomace. The temperature of loss of 5% of the material is 101 °C, due to the evaporation of the easily volatile substances, including water, as the graph shows for the filler before drying. According to the literature, after water is released from the filler, the decomposition process of lignin (160–500 °C), hemicellulose (220–315 °C) and cellulose (315–400 °C) is started [38,39]. The DTG curve contains three characteristic points, T1 with a maximum at 231 °C and a mass loss in the temperature range of 120–245 °C equal to 13%, mainly related to the thermal decomposition of hemicellulose, T2 with a maximum at 330 °C and a mass loss in the temperature range 245–375 °C equal to 36% related mainly to the decomposition of cellulose, and T3 with a maximum at 404 °C and a mass loss in the temperature range 375–500 °C equal to 13% related mainly to the decomposition of lignin and the decomposition of aromatic compounds. The residue after burning the plant filler at 800 °C was 25%.

**Figure 4:**
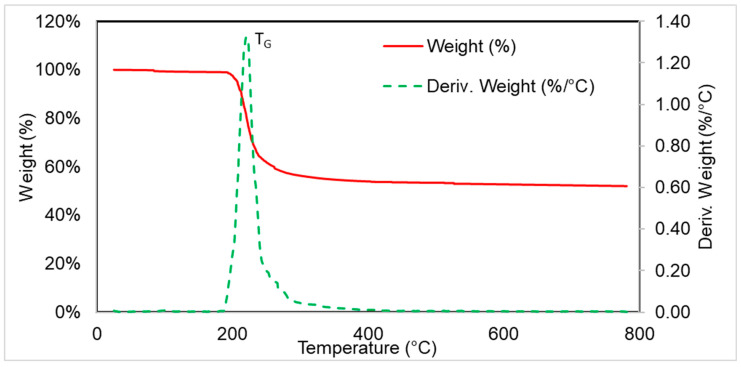
TG and DTG thermograms of expandable graphite.

The thermal degradation process of expandable graphite proceeded in one stage (Figure 4), with a maximum at T_G_ = 220 °C and a weight loss of 44% in the temperature range 180–300 °C. The residue after combustion at 800 °C was 53%.

**Figure 5:**
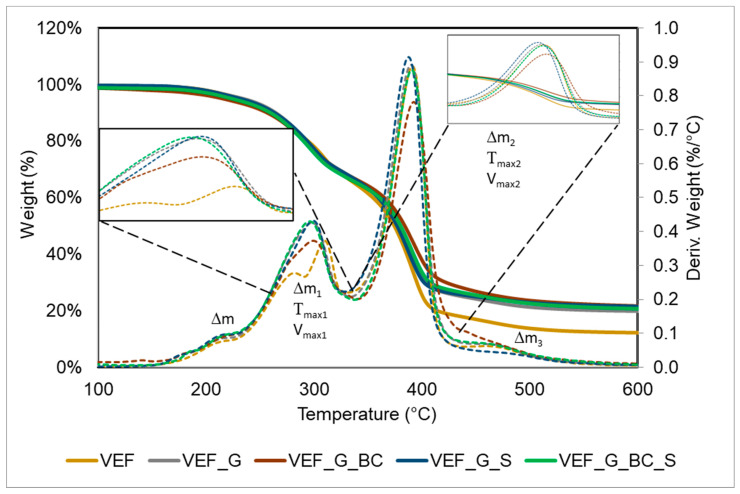
TG and DTG thermograms of foams.

Based on the curves of mass change as a function of temperature (TG), the temperature of loss of 5% of the mass of the polyurethane materials (T5%) and the residue at 600 °C (P600) were determined, as well as the mass changes corresponding to the subsequent stages of degradation (Δm, Δm1, Δm2, Δm3). The curves of the derivative of mass change as a function of temperature (DTG) were used to determine the maximum degradation rates in stage 2 (Vmax1) and stage 3 (Vmax2) and the temperatures at which these rates were reached (Tmax1 and Tmax2, respectively). The results are summarised in Figure 5 and Table 4.

The thermal degradation process of the VEF reference material passes in multiple stages. Based on the literature review, it was determined which products degraded in the following stages [22,28,38,40,41]. The first changes in the DTG curve in the temperature range of 50–230 °C are associated with the release of volatile substances and the decomposition of urea groups in polyurethanes, corresponding to a mass loss Δm of 4.3%. In the next stage of thermal degradation associated with mass loss Δm1 = 29.7% in the temperature range 230–335 °C, urea and urethane groups in rigid segments are thermally degraded. During this stage, the VEF material exhibited two peaks at Tmax1 temperatures of 281 °C and 309 °C, corresponding to maximum degradation rates of Vmax1 = 0.33%/°C and 0.43%/°C, respectively. The band in the 335–430 °C temperature range showed a maximum degradation rate of Vmax2 = 1.06%/°C at Tmax2 = 390 °C, which was formed by thermal degradation of the elastic segments. The weight loss of the sample at this stage is Δm2 = 47.7%. In the last stage of thermal degradation, contained in the 430–600 °C range, where the mass loss is Δm3 = 6.3%, pyrolysis of the combustion residue from earlier stages occurs. The combustion residue for the reference material at 600 °C is P_600_ = 11.9%.

The introduction of expandable graphite (VEF_G) into the reference system caused changes in the course of the second stage of thermal degradation contained in the temperature range 230–335 °C, as indicated by a signal with one maximum on the DTG curve with Tmax1 = 299 °C and Vmax1 = 0.43%/°C, which is due to the formation of carbonisation limiting the burning process of the material, also indicated by a decrease in the mass loss of Δm1 and Δm2, and a marked increase in P600 to ~20%. The observed changes result from thermal degradation of the graphite used, whose decomposition occurs in the temperature range of 180–300 °C. Modification of the VEF_G system with blackcurrant pomace (VEF_G_BC) did not affect the DTG curves, but resulted in a slight decrease in mass loss Δm1 and Δm2, and caused an increase in Δm3 and P600 to ~22%. These changes are due to the chemical structure of the natural fillers, particularly the proportion of lignin and cellulose capable of forming char in the burning process, as described in the work [34]. No significant effect of SOLKANE^®^ 365/227 was observed on the results of thermogravimetric analysis of foams.

### 3.4. Flammability Analysis of Materials

**Figure 6:**
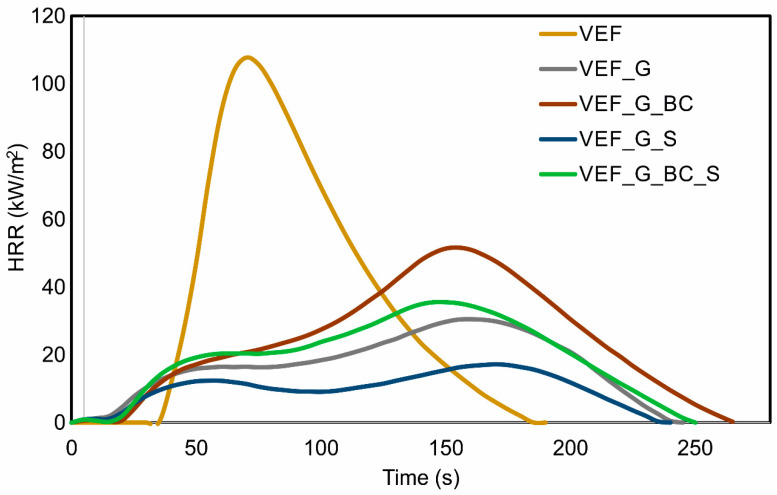
Representative heat release rate curves obtained for analysed materials.

The values of key parameters of the combustion process of materials obtained from the test carried out using a cone microcalorimeter are summarised in Table 5. The table also lists the FIGRA (Fire Growth Rate Index) values. These are defined as the ratios of the peak HRRmax to the time it is achieved. The smaller the FIGRA, the more time it takes to evacuate and provide the necessary firefighting equipment. Figure 6 shows representative heat release rate curves as a function of time for the materials analysed. Flammability ratings were also determined for the materials following UL-94, and the limiting oxygen index was determined (Table 6).

The flammability analysis indicates that introducing expandable graphite into the reference system alters the materials’ combustion behaviour. The single signal for VEF with pHRRmax = 107.7 kW/m^2^ observed after a time of 70 s is replaced by a curve with two maxima occurring after 50 s and 160 s, and pHRRmax for this material is reduced by 72%, reaching a value of 30.5 kW/m^2^. The curve obtained for the VEF_G material by its shape corresponds to curves with intumescent additives and suggests the formation of an insulating layer on the material’s surface, protecting it from the heat flux. A similar combustion process for foams with expandable graphite was presented in the work of Oliwa and co-authors [28]. Introducing blackcurrant pomace (VEF_G_BC) into the systems increases pHRRmax, but this value is 52% lower compared to the unmodified VEF material. Introducing the SOLKANE^®^ 365/227 additive into the VEF_G and VEF_G_BC blends resulted in a decrease in pHRRmax, which may be due to the share of fluorine in the chemical composition of this additive. SOLKANE^®^ 365/227 is a blend of 1,1,1,3,3-pentafluorobutane and 1,1,1,2,3,3,3-heptafluoropropane. The possibility of reducing the flammability of polymeric materials using fluorine compounds is described in numerous patents [42,43,44]. Flame retardancy using halogen compounds is among the chemical methods where halogen compounds act as free radical scavengers [45]. The compound SOLKANE^®^ 227 is an extinguishing agent in aviation firefighting systems. The analysis results show that the material modified with blackcurrant pomace, graphite and SOLKANE^®^ 365/227 has a pHRRmax value 67% lower than the unmodified VEF material. The total heat released during combustion (THR) also changes with the change in foam additives. In all cases, introducing additives to the VEF matrix resulted in lower THR values. The observed changes correlate with changes in pHRR.

The analysed materials were characterised by a relatively short time to ignition. Introducing GE, BC and S additives reduced the time required to ignition (Ti) for the materials. The short time to ignite polyurethane foams is due to the structure of the foamed materials and their low thermal conductivity. A single-cell strut acts like a thermally thin material and reaches its ignition temperature much faster than a bulk material because heat conduction within the material is limited. Upon ignition, a thin, liquid layer of intermediate pyrolysis products forms. This layer travels from the top to the bottom of the specimen as the foam sample collapses, leading to a pool fire [46]. The use of expandable graphite increased the proportion of combustion residue (Rc) as it formed a char layer on the material’s surface during combustion, limiting the access of heat fluxes. The introduction of blackcurrant pomace into the mixtures with G and G_S resulted in a decrease in the proportion of combustion residue, which may be due to the mass loss in the last stage of degradation Δm3 observed in the curves obtained by thermogravimetric analysis (Figure 3, Table 4). Applying SOLKANE^®^ 365/227 did not significantly affect the Rc.

The Fire Growth Rate Index for all graphite-modified foams is significantly lower than for VEF. The introduction of graphite causes a reduction in FIGRA from 1.54 for VEF foam to 0.19 for VEF_G foam, i.e., by approximately 88%. The addition of blackcurrant filler to VEF_G causes an increase in FIGRA. A favourable reduction in FIGRA results from the introduction of the SOLKANE^®^ 365/227 blowing agent into the VEF_G and VEF_G_BC mixture to values of 0.10 (VEF_G_S) and 0.24 (VEF_G_BC_S), respectively. This change also confirms the flame-retardant effect of this blowing agent.

Flammability tests conducted following UL-94 and a limited oxygen index (LOI) analysis also indicate that foams modified with G, BC and S additives have higher flame resistance than the reference material. The reference material burned completely in the test conducted on vertical samples (flammability class V) and horizontal samples (flammability class HB) and does not meet the requirements necessary to qualify for the flammability classes mentioned above. The high flammability of the material is also indicated by the low LOI value of 20.6%. This is the standard value obtained for flexible polyurethane foams, qualifying the material for the group of flammable plastics (materials are considered flammable when their oxygen index in an air atmosphere is lower than 21%) [47]. The flammability of flexible polyurethane foams is related to their developed pore surface and their high air permeability through their open-cell structure. The introduction of expandable graphite (VEF_G) into the reference system increased the fire resistance of the foams, leading to a flammability class of HB40 and an increase in the oxygen index to 27.5%. The introduction of blackcurrant pomace into VEF_G foam led to an even higher increase in the fire resistance of the materials, resulting in a V-0 rating in the vertical test and HB40 in the horizontal test (VEF_G_BC), as well as an increase in the oxygen index to a value of 27.9%. The introduction of SOLKANE^®^ 365/227 into the graphite system (VEF_G_S) had an equal effect on the increase in flammability of the materials. Modification of the system with graphite with both blackcurrant pomace and SOLKANE^®^ 365/227—VEF_G_BC_S foam led to a decrease in the flammability class of the material to HB40 and a slight decrease in the oxygen index to a value of 26.8%, which may be due to changes in the structure of the material after the introduction of additives, in particular, an increase in the proportion of large pores that facilitate the spread of flames (Figure 1 and Figure 2) [28].

**Figure 7:**
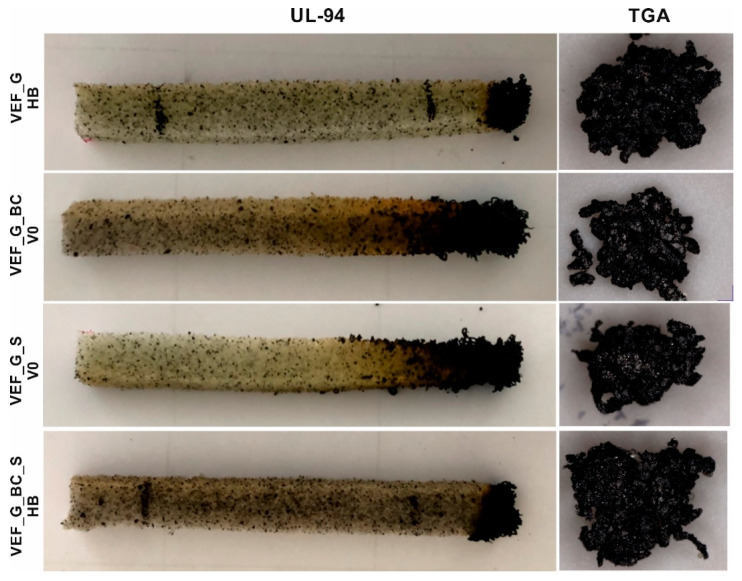
Images after conducting UL-94 and TGA test.

Figure 7 shows photos of foam composite samples subjected to flammability testing per UL-94 and photos of char obtained from thermogravimetric analysis of the materials conducted in an air atmosphere from room temperature up to 1000 °C. The reference material samples burned completely in both tests. The images of G, BC and S-modified materials indicate the dominant effect of expandable graphite in reducing the burning of materials. As indicated by literature sources, the interaction of expandable graphite is mainly in the condensed phase [48]. In the initial phase, the graphite acts to shield thermal radiation from an excessive temperature rise. Then, due to the release of gases, the graphite swells and forms an insulating coating. Finally, the extruded graphite forms a refractory shield against oxygen and flame access to the deeper layers of the material, which also acts as a barrier to heat and mass transfer from the polymer to the heat source, thus preventing further decomposition of the material [49,50].

### 3.5. Differential Scanning Calorimetry Studies of Materials

**Figure 8:**
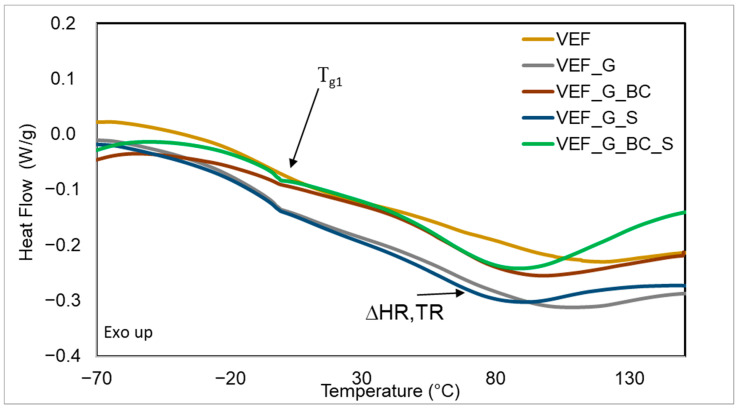
DSC curves (C1) of the developed materials.

**Figure 9:**
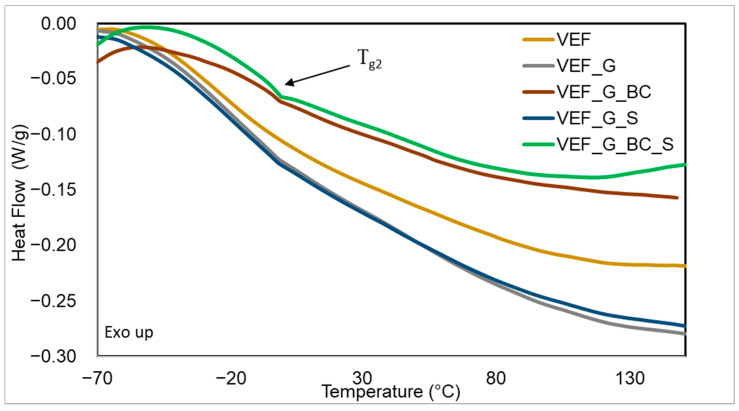
DSC curves (C2) of the developed materials.

Based on the DSC thermogram of the foams, it was observed that during the first heating (C1), a bend typical for the glass transition temperature (Tg1) in the soft phase of polyurethane appeared on the curves, along with an endothermic peak from the extremum in temperature (TR) in the temperature range of 80–110 °C and an enthalpy of transformation ∆HR resulting from the order–disorder transformation in the hard phase of PU (Figure 8). During the second heating of the sample (C2), only the bend typical for the glass transition temperature of the soft phase (Tg2) was observed (Figure 9). Table 7 summarises the determined Tg1, Tg2, TR and ∆H.

The results of the DSC analysis indicate that the use of expandable graphite caused a slight increase in Tg1 and Tg2, suggesting a limitation in the mobility of the flexible segments of the soft phase due to the introduction of this powdered additive [38]. An increase in ∆HR was also observed after the addition of expandable graphite. This indicates that a greater amount of the ordered hard phase bound by hydrogen bonds formed during the phase separation process in these materials [28]. The use of blackcurrant pomace in the production of PU composites did not affect the glass transition temperature of the materials and caused a slight increase in ∆HR. The use of SOLKANE^®^ 365/227 had no impact on the described values. Adding fillers to the VEF system caused a shift in TR towards lower values. The analysis of the foams in the heating–cooling–heating cycle indicates high stability of the glass transition temperatures of the foams, as no significant differences were observed in Tg1 and Tg2 values.

### 3.6. The Chemical Constitution Analysis of the Materials

**Figure 10:**
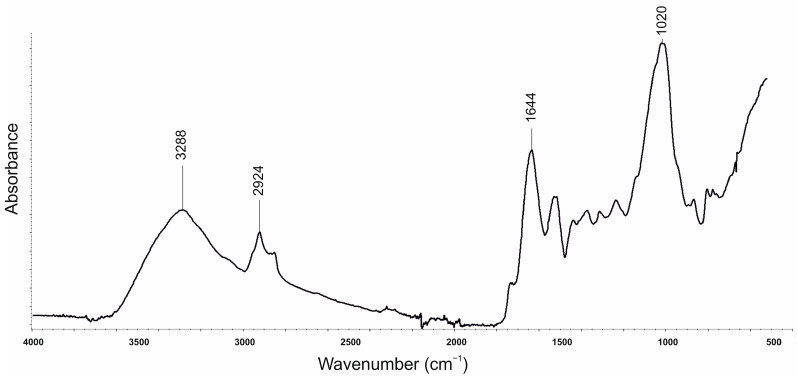
Spectra FTIR of blackcurrant pomace.

**Figure 11:**
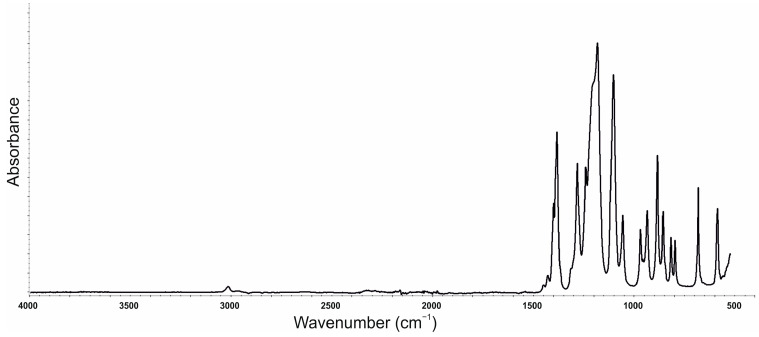
Spectra FTIR of SOLKANE^®^ 365/227.

**Figure 12:**
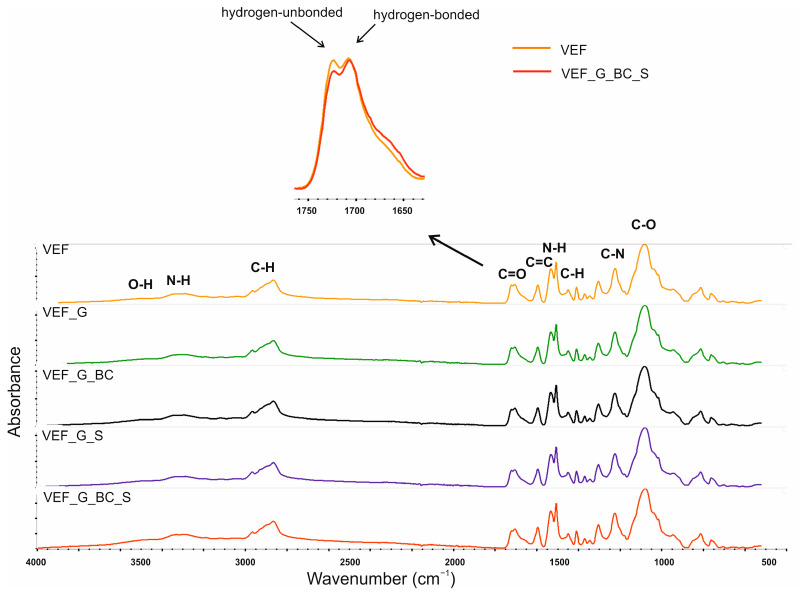
FTIR spectra of the analysed polyurethane materials.

The FTIR spectroscopy was conducted for blackcurrant pomace (Figure 10), SOLKANE^®^ 365/227 (Figure 11) and PU composites (Figure 12) in the ATR mode. The results for the composites are summarised in Table 8.

The results of the analysis of the FTIR spectra performed for blackcurrant pomace indicate a broad band with a maximum at a wavenumber of 3288 cm^−1^ due to the presence of hydroxyl groups in lignin, hemicellulose and cellulose. We also observed bands that originate from asymmetric and symmetric stretching vibrations of the -CH group with a maximum of 2924 cm^−1^, and a band with a maximum wavenumber at 1644 cm^−1^ due to the presence of a carbonyl group (C=O) mainly associated with hemicellulose and due to the presence of C=C _aromatic_, C=C _stretching_ associated with lignin. Moreover, we observed a band with a maximum of 1020 cm^−1^ (C-O stretching) as specific for the vibrations derived from cellulose and hemicellulose [51].

The FTIR spectra obtained for SOLKANE^®^ 365/227 correspond to those available in the literature for hydrofluorocarbons with characteristic bands in wavenumbers 1500–1000 cm^−1^ [52].

The results of the FTIR spectra analysis included in the publication indicate that bands characteristic of expandable graphite, such as C=C, occur in the 1340–1700 cm^−1^ range. The absorption peak at 1118 cm^−1^ corresponds to C-OH stretching and OH bending vibrations, and the peaks at 2921 cm^−1^ and 2852 cm^−1^ originate from C-H stretching vibrations. The peaks at 1580 cm^−1^ and 1636 cm^−1^ are due to the stretching vibration of the carboxylic group.

The FTIR spectra of the produced PU materials showed characteristic bands for polyurethanes, indicating the course of the planned synthesis. The broadband in the wavenumber range of 3600–3400 cm^−1^ originates from the symmetrical stretching vibrations of -OH groups from polyols, whose presence is due to the use of an isocyanate index below 100. In the 3400–3200 cm^−1^ range, there is a broad peak resulting from the asymmetric and symmetric stretching vibrations of the -N-H group present in urethane and urea derivatives. The bands with maxima at wavenumbers 1536 cm^−1^ and 1509 cm^−1^ indicate this group’s bending vibrations. The bands with maxima at wavenumbers 2969–2968 cm^−1^ and 2868 cm^−1^ originate from the asymmetric and symmetric stretching vibrations of the CH_3_ and CH_2_ groups. The bands with maxima at 1452 cm^−1^ (CH_3_) and 1373 cm^−1^ (CH_2_) result from the asymmetric and symmetric bending vibrations, and bands with a maximum of 1306 cm^−1^ originate from stretching vibrations. Multiplet signals in 1770–1620 cm^−1^ indicate the presence of C=O carbonyl groups in urethane and urea structures. The signal maxima at 1722 cm^−1^ and 1708 cm^−1^ correspond to the stretching vibrations in nonbonded and hydrogen-bonded carbonyl groups, respectively. Signals with a maximum of 1597 cm^−1^ originate from multiple stretching aromatic ring vibrations. The bands with a maximum wavenumber of 1227–1226 cm^−1^ originate from stretching vibrations of the C-N group. The signal with a maximum wavenumber value of 1085–1083 cm^−1^ originates from stretching vibrations of the C-O group forming the elastic segments of polyurethane. No signal with a maximum of 2270 cm^−1^ was observed on the FTIR spectra of the foams, indicating that the NCO groups were completely over-reacted [6,22,28,53,54,55].

Differences in the shape of the multiplet signal in the range of wavenumbers 1760–1630 cm^−1^ on the FTIR spectra of foams modified with blackcurrant pomace were observed, consisting of a change in the intensity of the components derived from the stretching vibrations of the carbonyl groups of the urethane grouping bound by hydrogen bonds and unbound (Figure 12). The results of the analysis show a higher proportion of bound carbonyl groups in BC-modified foam samples (VEF_G_BC; VEF_G_BS_S), which indicates a higher degree of phase separation of the rigid segments in these foams and may be due to the rearrangement of the rigid segments related to the flexible segments caused by the introduction of BC filler. An increase in the degree of phase separation of the rigid segments in PU composites after the introduction of natural filler was also reported in the paper by Zieleniewska et al. [56]. Modifying the foams with additives in the form of expandable graphite and also SOLKANE^®^ 365/227 did not cause changes in the position of signals characteristic of polyurethanes, indicating that this was a physical modification [6]. Additional signals were also not observed on the composites’ FTIR spectra.

### 3.7. Analysis of the Physico-Mechanical Properties of Materials

A new system of flame-retardant additives and biocomponents requires verification of the basic properties of these materials, i.e., density, compressive strength and resilience. The properties determined as part of the work are summarised in Table 9.

The results of the analysis of the physico-mechanical properties show an increase in the apparent density of the foams after the introduction of solid additives, by 7% after the introduction of expandable graphite into the VEF and by 16% after the introduction of 30 php blackcurrant pomace into the VEF_G system, which is due to the higher density of solid additives compared to the apparent density of the PU matrix (Table 9) [57]. The use of an additional blowing agent in the form of SOLKANE^®^ 365/227 resulted in a 21% decrease in the apparent density of the VEF_G material, but the introduction of 30 php of currant pomace into the system resulted in a significant 44% increase in the apparent density of this foam. Similar relationships were also observed in the works of other authors [37].

An important indicator of the mechanical resistance of foams used in practical applications is their compression set, determined at a compression to 50% of the initial height. It is assumed that the permanent deformation value for elastic and viscoelastic foams should not exceed 15% [45]. The analysis result of the compression set of the developed foams indicates that all the produced materials fulfil the requirements for viscoelastic foams (Table 9) [57]. The use of solid additives in the form of expandable graphite and blackcurrant pomace had a favourable effect on the mechanical resistance of the foams, reducing the value of the compression set. The introduction of SOLKANE^®^ 365/227 (VEF_G_S) increased strain values, but modification of the system with a filler of plant origin made it possible to reduce the adverse effect (VEF_G_S_P).

A characteristic feature of viscoelastic foams is low resilience, not exceeding 20% [45]. The analysis results show that all the foams studied had a resilience corresponding to this group of materials (Table 9). No significant changes in this property were observed after solid additives G and BC were introduced, as well as additional blowing agent S.

The hardness of the foam is measured by stresses at 40% compression of the sample height. It is often correlated with the density of flexible foam and its chemical composition. However, it was observed that, despite the apparent density increase, the foams’ hardness decreased in VEF_G_BC and VEF_G_BC_S. One of the possibilities could be due to the hydrophilic character of blackcurrant pomace, which is incompatible with the polyurethane matrix, weakening the material’s compressive strength. Sample VEF_G_S, with SOLKANE^®^ 365/227 blowing agent, resulted in over a 40% reduction in the CV40 parameter.

The SAG comfort factor was determined by dividing the resulting stresses of the sample compression at 65% and 25% of the probe height. The SAG parameter is used to evaluate cushioning in the furniture industry. The reference foam had a SAG factor of 2.44. The addition of graphite (VEF_G sample) caused an increase in the SAG parameter to 2.95. Sample with graphite and SOLKANE^®^ 365/227 (VEF_G_S) had a SAG factor of 3.02, which is very similar, considering the measurements’ standard deviation. Samples with blackcurrant pomace (VEF_G_BC, VEF_G_BC_S) exhibited increased values of SAG comfort factor, which might be beneficial in case of potential uses of modified material. The value of the SAG factor in the VEF_G_BC_S sample is twice as high as for the VEF_G_BC sample, which indicates that some synergistic effect of SOLKANE^®^ 365/227 blowing agent with blackcurrant pomace occurred.

**Figure 13:**
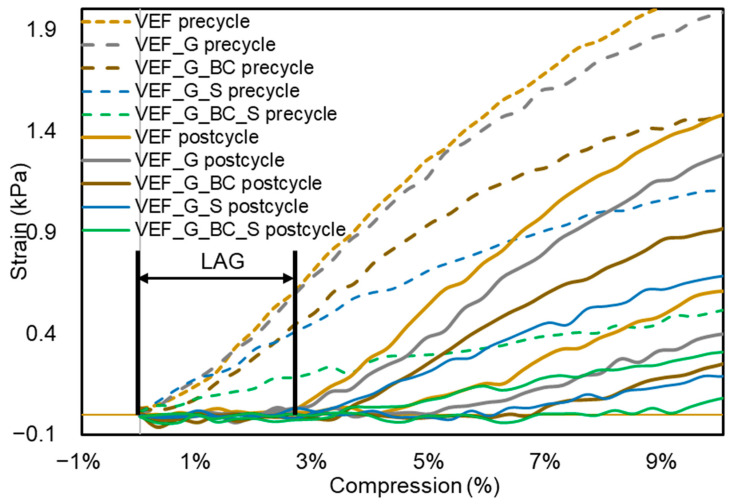
Strain–compression correlation charts for the analysed foams.

In Figure 13, a certain lag can be observed. A typical viscoelastic behaviour proves that the additives researched under the described conditions do not affect the foam’s viscoelastic properties.

The graph’s dashed line shows the first compression cycle, the precycle. This is when the top plate of the mechanical compression tester touches the sample and starts to compress it. After the first cycle, the compression tester relaxes the sample and then compresses it. The lag between the precycle and postcycle shows the viscoelastic behaviour of the foams, which is shown by the very slow return. The bigger the lag, the slower movement the foam provides. In this test and also the resilience tests, it was proven that viscoelastic properties had not been changed significantly. 

## 4. Conclusions

The results described indicate that composites of viscoelastic polyurethane foams with limited flammability characterised by a high content (30 php) of renewable raw material in the form of ground blackcurrant pomace, which is a waste from fruit processing, were successfully developed. The flammability of the materials was reduced using expandable graphite in a proportion of 15 php. The results show that the viscoelastic foam composite containing 30 php of blackcurrant pomace and 15 php of expandable graphite has a pHRRmax 52% lower than that of the reference material. The additional use of a blowing agent SOLKANE^®^ 365/227 in a proportion of 10 php enhanced the flame-retardant effect of the materials, resulting in a 67% reduction in pHRR compared to the reference material. However, compared to the material made with expandable graphite and solkane, the foam made with G, S and BC had more than twice the flammability. Introduction of the blackcurrant pomace, expandable graphite and SOLKANE^®^ 365/227 additives to the reference material resulted in the preparation of composites with flammability classes HB40 and V-0 according to UL-94, while the reference material burned completely during the test.

Moreover, the limiting oxygen index increased from 21% for the reference material to 26–28% for the foam composites. The thermal properties of the developed viscoelastic composites were changed due to the introduction of the G, BC and S additives, as demonstrated by the TGA and DSC studies, which indicated an increased glass transition temperature of the materials due to changes in the mobility of the flexible polymer segments. The effect of blackcurrant pomace on the increase in the degree of phase separation of the materials was also observed. While introducing solid additives (G and BC) increased the apparent density, SOLKANE^®^ 365/227 blowing agent allowed for its reduction. The produced materials were characterised by a low compression set and low resilience. The material produced using G, S and BC had the highest SAG factor. The results presented in this article demonstrate a novel approach to producing viscoelastic polyurethane foam composites using waste vegetable filler, which is a significant step towards sustainable development and a circular economy. The strength of the solution is the significant reduction in flammability of the materials to the reference material while using renewable raw materials, further reducing production costs. While the introduction of additives may lead to an increase in the density of the foams, this simultaneously improves their mechanical properties, which increases the application potential in more demanding applications.

Due to their flame-retardant properties and mechanical durability, the developed foam composites present a high potential for application across various industrial sectors. In the automotive and aerospace industries, where lightweight, flame-resistant materials are critical, these biocomposites may serve as viable materials for interior components, including seat padding and sound-absorbing elements. Their viscoelastic properties also make them particularly suitable for bedding and furniture applications, where a balance of comfort, low resilience and enhanced fire safety is essential. The consumer electronics sector could also benefit from these foams as protective casings, padding and enclosures requiring impact absorption and fire resistance. By integrating renewable, cost-effective waste materials, these bio-based foams contribute significantly to sustainable production practices and support the principles of the circular economy.

In conclusion, foam with blackcurrant, graphite and SOLKANE^®^ 365/227 offers an attractive compromise between environmental performance, mechanical properties and reduced flammability, making it an interesting option in sustainability and modern applications.

## Figures and Tables

**Table 1:** Differences in the composition of the materials manufactured.

Sample	Additives in Polyols, php			Additives in Foam Substrates, wt. %			
	BC	G	S	BC	G	S	Polyurethane Substrates
VEF	-	-	-	-	-	-	100
VEF_G	-	15	-	-	8.7	-	91.3
VEF_G_BC	30	15	-	14.8	7.3	-	77.9
VEF_G_S	-	15	10	-	8.2	5.5	86.3
VEF_G_BC_S	30	15	10	14.1	7.1	4.7	74.1

**Table 2:** Characteristic times of the foaming process.

Sample	Start Time, s	Growth Time, s
VEF	25	240
VEF_G	35	200
VEF_G_BC	45	220
VEF_G_S	30	190
VEF_G_BC_S	35	240

**Table 3:** Size intervals and average pore sizes in foams.

	Size Intervals (µm)	Average Pore Sizes (µm)
VEF	327–2909	1354
VEF_G	411–1562	994
VEF_G_BC	352–2119	1407
VEF_G_S	515–2789	1724
VEF_G_BC_S	753–2980	1939

**Table 4:** The results of thermogravimetric analysis.

	T5% (°C)	Δm (%), 50–230 °C	Tmax1 (°C), (Vmax1, %/°C)	Δm1 (%), 230–335 °C	Tmax2 (°C), Vmax2, %/°C	Δm2 (%), 335–430 °C	Δm3 (%) (430–600 °C)	P600 (%)
VEF	235	4.3	281 (0.33) 309 (0.46)	29.7	390 (1.06)	47.7	6.3	11.9
VEF_G	234	4.3	299 (0.43)	28.4	390 (0.89)	41.5	5.8	19.8
VEF_G_BC	216	6.0	299 (0.37)	26.4	392 (0.78)	38.4	7.3	21.6
VEF_G_S	231	4.6	299 (0.43)	27.9	387 (0.91)	41.2	4. 6	21.5
VEF_G_BC_S	219	5.4	300 (0.42)	26.6	391 (0.88)	39.0	6.0	21.4

**Table 5:** The results of the flammability analysis of materials.

Sample	Ti (s)	pHRR (kW/m^2^)	Time to pHRR (s)	pHRRmax (kW/m^2^)	Time to pHRRmax (s)	THR (MJ/m^2^)	Rc (%)	FIGRA (kW/m^2^·s)
VEF	24	-	-	107.7	70	8.5	9.7	1.54
VEF_G	7	15.9	50	30.5	160	4.1	37.8	0.19
VEF_G_BC	13	17.2	50	51.6	155	8.2	29.0	0.33
VEF_G_S	8	12.2	50	17.2	170	3.2	38.6	0.10
VEF_G_BC_S	10	19.2	50	35.6	150	6.2	32.7	0.24

**Table 6:** Results of UL-94 flammability rating and limiting oxygen index analysis.

Sample	UL-94 Classification	LOI, %
VEF	- ^1^	20.6
VEF_G	- ^2^, HB40	27.5
VEF_G_BC	V0, HB40	27.9
VEF_G_S	V0, HB40	27.8
VEF_G_BC_S	- ^2^, HB40	26.8

^1^ The material burned completely in the vertical and horizontal test conducted in accordance with the UL-94 standard. ^2^ The material burned completely in a vertical test conducted following UL-94.

**Table 7:** The results of the DSC curve analysis of the examined materials.

	Tg1 (°C)	∆HR (J/g)	TR (°C)	Tg2 (°C)
VEF	−7.0	18.3	110.2	−8.2
VEF_G	−3.2	30.9	97.4	−4.5
VEF_G_BC	−3.3	33.1	93.9	−3.4
VEF_G_S	−3.5	31.0	82.1	−4.1
VEF_G_BC_S	−3.5	36.2	85.2	−3.6

**Table 8:** Wavenumbers and corresponding characteristic bands determined for foams.

VEF	VEF_G	VEF_G_BC	VEF_G_S	VEF_G_BC_S	Bond (Vibration)
Wavenumbers [cm^−1^]					
3600–3400					O-H (stretching)
3400–3200					N-H (stretching)
2969	2968	2968	2969	2968	C-H (asymmetric stretching)
2868	2868	2868	2868	2868	C-H (symmetric stretching)
1722	1722	1722	1722	1722	C=O (stretching/unbounded)
1708	1708	1708	1708	1708	C=O (stretching/bounded)
1597	1597	1597	1597	1597	C=C (stretching)
1536	1536	1536	1536	1536	N-H (bending)
1509	1509	1509	1509	1509	N-H (bending)
1452	1452	1452	1452	1452	C-H (deformation, asymmetric)
1373	1373	1373	1373	1373	C-H (deformation, symmetric)
1306	1306	1306	1306	1306	C-H (stretching)
1226	1226	1227	1226	1227	C-N (stretching)
1085	1084	1085	1085	1083	C-O (stretching)

**Table 9:** Analysis of apparent density, compression set and resilience.

Sample	Apparent Density (kg/m^3^)	Compression Set (%), 50%, 22 h, 70 °C ^1^	Resilience (%)	Hardness CV40, [kPa]	Comfort Factor—SAG, Units
VEF	48 ± 1	4.0 ± 0.0	7.2 ± 0.8	0.94 ± 0.01	2.44 ± 0.07
VEF_G	52 ± 1	2.7 ± 1.9	7.0 ± 0.9	0.90 ± 0.01	2.95 ± 0.09
VEF_G_BC	60 ± 2	2.7 ± 1.9	6.9 ± 0.8	0.64 ± 0.06	3.14 ± 0.49
VEF_G_S	41 ± 1	6.7 ± 1.9	7.0 ± 0.7	0.54 ± 0.04	3.02 ± 0.08
VEF_G_BC_S	58 ± 1	2.7 ± 1.9	8.3 ± 1.0	0.47 ± 0.02	6.07 ± 0.24

^1^ Conditions for conducting the analysis.

## Data Availability

The original contributions presented in the study are included in the article, further inquiries can be directed to the corresponding author.
